# Onchocerciasis Control: Vision for the Future from a Ghanian perspective

**DOI:** 10.1186/1756-3305-2-7

**Published:** 2009-01-21

**Authors:** Mark J Taylor, Kwablah Awadzi, María-Gloria Basáñez, Nana Biritwum, Daniel Boakye, Boakye Boatin, Moses Bockarie, Thomas S Churcher, Alex Debrah, Geoffrey Edwards, Achim Hoerauf, Sabine Mand, Graham Matthews, Mike Osei-Atweneboana, Roger K Prichard, Samuel Wanji, Ohene Adjei

**Affiliations:** 1 Molecular and Biochemical Parasitology, Liverpool School of Tropical Medicine, Liverpool, UK; 2 Onchocerciasis Chemotherapy Research Centre (OCRC), Hohoe Hospital, Hohoe, Ghana; 3 Department of Infectious Disease Epidemiology, Imperial College, London, UK; 4 Lymphatic Filariasis Elimination Programme, Health Research Unit, Ghana Health Service, Accra, Ghana; 5 Noguchi Memorial Institute for Medical Research, University of Ghana, Legon, Accra, Ghana; 6 TDR, World Health Organisation, Geneva, Switzerland; 7 Centre for Neglected Tropical Diseases (incorporating the Lymphatic Filariasis Support Centre), Liverpool School of Tropical Medicine, Liverpool, UK; 8 Kumasi Centre for Collaborative Research (KCCR), Kwame Nkrumah University of Science and Technology (KNUST), Kumasi, Ghana; 9 Institute for Medical Microbiology, Immunology & Parasitology, University of Bonn, Bonn, Germany; 10 Department of Biological Sciences, Imperial College of Science, Technology and Medicine, London, UK; 11 Institute of Parasitology, McGill University, Québec, Canada; 12 Research Foundation for Tropical Diseases and the Environment (REFOTDE), Buea, Cameroon

## Abstract

Since 1987 onchocerciasis control has relied on the donation of ivermectin (Mectizan^®^, Merck & Co., Inc.) through the Mectizan Donation Programme. Recently, concern has been raised over the appearance of suboptimal responses to ivermectin in Ghana – highlighting the potential threat of the development of resistance to ivermectin. This report summarises a meeting held in Ghana to set the research agenda for future onchocerciasis control. The aim of this workshop was to define the research priorities for alternative drug and treatment regimes and control strategies to treat populations with existing evidence of suboptimal responsiveness and define research priorities for future control strategies in the event of the development of widespread ivermectin resistance.

## Introduction

Since 1987 onchocerciasis control has relied on the donation of ivermectin (Mectizan^®^, Merck & Co., Inc.) through the Mectizan Donation Programme (MDP). This programme is rightly regarded as a model paradigm for mass treatment control programmes [1]. The 20^th ^anniversary of MDP was an opportunity to reflect on the successes the programme has achieved [2]. As the African Programme for Onchocerciasis Control (APOC) enters the phasing-out period of the programme (2008–2015) a number of ongoing and future challenges face the sustained delivery of onchocerciasis control by national programmes. While attention on the problems presented by 'fragile states', poor primary health care systems and co-infection with *Loa loa *are evident [2-4] – the role and priorities for research activities to address complementary and alternative control strategies have received less attention. This is particularly important in view of the conclusion that onchocerciasis in Africa cannot be eliminated with existing tools [5]. To address this issue a meeting was held in Ghana in April 2008. The aim of this meeting was to review the current status of onchocerciasis control in Ghana in light of evidence of poor-responsiveness to ivermectin, define the research priorities for alternative drug and treatment regimes and control strategies to treat populations with existing evidence of suboptimal responsiveness and define research priorities for future control strategies in the event of the development of widespread ivermectin resistance.

### The current status of onchocerciasis control in Ghana

In Ghana, onchocerciasis is endemic in 9 out of 10 regions. About 3204 communities from 66 districts are endemic in these regions. Some 247 of these communities in Brong Ahafo and Ashanti regions have been designated as Special Intervention Zones (SIZ), which are areas of hyperendemicity, within the Pru River basin that serve as the focus of Community-Directed Treatment with Ivermectin (CDTI). The total at risk population for onchocerciasis is about 3.2 million.

Ivermectin distribution in Ghana started with the use of mobile teams in 1987, with CDTI introduced in 1998. Since the devolution of onchocerciasis control from the former-OCP (Onchocerciasis Control Programme) to Ghana (under APOC), the national programme has been monitoring recrudescence through entomological and epidemiological surveillance activities. Ivermectin treatment started in lymphatic filariasis and onchocerciasis co-endemic areas in 2001 and has undergone a gradual up-scaling to cover 61 endemic districts by 2005. From 2002 to 2007 3.4 million people were treated through CDTI with coverage ranging from 48.4 to 79.1%. Since 2006 onchocerciasis control has been implemented in the context of the Neglected Tropical Diseases Control Programme (NTDCP). Implementation of NTDCP started in April 2007 on a pilot basis in 5 regions of Ghana.

Results from the entomological and epidemiological surveillance activities have indicated fly infectivity levels and infection in humans that require improved programme attention. Results of surveys done in 2005, indicated high infectivity rates of 1.82 per 1000 parous flies, from the Asubende site within the Pru River basin. In 2006 results again showed high infectivity rates of 0.556 – 1.01 per 1000 parous flies from sites within the White Volta, Kulpawn, Anum and Pra River basins. Fly nuisance was significantly high in each of the surveyed sites.

In 2004 epidemiological surveys showed a microfilaria positive prevalence of 8.9% with 13 out of the 18 villages surveyed having prevalence of more than 5%. About 2.8% and 0.75% had visual impairment and total blindness respectively. About 9 of the 24 communities surveyed in 2006 had prevalence rates above 5% with the highest focus found in 3 sites in the Asante Akim district in Ashanti region. Community microfilarial load (CMFL) ranged from 0.56 – 2.89 mf per skin snip. A site within the Pru River basin produced CMFL of 0.98, which is above the threshold of 0.5 while results of other communities were below this threshold.

A number of factors may contribute to the trends observed from entomological and epidemiological surveys, including CDTI coverage, individual compliance and the possibility that the efficacy of ivermectin is compromised.

### Evidence for poor responsiveness and resistance to ivermectin

#### Evidence for Sub-optimal Responses to Ivermectin

Suboptimal response (SOR) of *Onchocerca volvulus *to ivermectin is manifested by responses that are incompatible with previous experience with multiple treatments. Critical to this definition is incontrovertible evidence of the consumption by the subject of the number of treatments under consideration. Additional factors are parasite exposure to adequate concentrations of ivermectin and control for the effects of ongoing transmission. SOR is usually suspected when the prevalence of and/or the intensity of infection by skin microfilariae are higher than expected and is confirmed by direct examination of the adult worms. Multiple treatments with ivermectin have marked effects on embryogenesis [6]. Quantitative estimates have ranged from an irreversible decline in microfilarial production of ~30% per treatment [7], a reduction in the productivity index of 83% [8] to arrest of development at the single cell stage [9]. Thus when the factors listed above are taken into account, the presence of normal reproductive activity in female worms exposed to multiple treatments is incompatible with a normal response.

An open case control hospital based study [10] enrolled 21 microfilaridermic and 7 amicrofilaridermic subjects from two river basins in Ghana with little risk of ongoing transmission and with documented consumption of ≥ 9 ivermectin treatments, and also 14 microfilaridermic ivermectin naïve subjects from a focus of continuing transmission. Detailed clinical ophthalmological and laboratory examinations were done. Ivermectin 150 μg/kg was then administered. A pharmacokinetic component determined drug exposure. Skin snipping and fly feeding were done before and at days 8, 90 and 365. Embryograms from adult female worms were examined at day 90. The study determined that the significant microfilaridermias despite multiple treatments with ivermectin were mainly attributable to the non-response of the adult female worms and not to inadequate drug exposure or other factors. This was confirmed in a 30-month follow up study [11]. Microfilariae in general remained sensitive to ivermectin.

In a two phase epidemiological study [12], 2501 individuals were randomly selected from 19 onchocerciasis endemic communities located in 3 river basins in Ghana. Very stringent criteria were met by the communities and detailed investigations provided incontrovertible evidence of the consumption of 6–18 rounds of ivermectin by the participants; one other community was ivermectin naïve. The microfilaria prevalence and community microfilarial load ranged from 2.2% to 51.8%, and 0.06 microfilariae per snip to 2.85 microfilariae per snip, respectively. Assessment of skin microfilariae 30 days after 150 μg/kg of ivermectin treatment showed 100% clearance in more than 99% of subjects. However, re-snipping of 342 initially skin snip positive participants from 10 communities at days 90 and 180 during the second phase revealed an usually high repopulation rate at day 90 that was confirmed at day 180 in 4 out of 10 communities. This finding suggests that the adult female worms had not responded to the known suppressive effects of the multiple treatments of ivermectin administered previously.

The Ghana studies (either conducted with little risk of new infections [10,11] or with ongoing transmission [12]) yielded similar results and demonstrate that ivermectin remains a potent microfilaricide but that non-responsive female adults may be present in some communities. This is also suggested by the much more rapid return of skin microfilariae at 4–6 months in a subgroup of subjects treated with a single dose of ivermectin and living in a non-endemic area [13].

#### Genetic evidence of resistance to ivermectin

Drug resistance has been defined as a loss of the normal response to treatment and is heritable [14]. Ivermectin affects both the microfilariae, removing them from the skin, and the adult worms, inhibiting their reproduction for many weeks. Because of these various and prolonged effects, and because ivermectin activity involves host immunity, it is not reliable to assess efficacy *in vitro*. Phenotypic assessment of resistance needs to consider both skin microfilarial loads (repopulation of the skin by microfilariae), and worm fertility (by embryogram). A meta-analysis was conducted of both these outcomes after single-dose ivermectin following a systematic review of early clinical and field trials and fitted a mathematical model to the data [15]. Results were compared with those obtained in a study of 10 repeatedly treated communities in Ghana (with >10 annual treatments) [12]. This study indicated continued high microfilaricidal activity of ivermectin but suggested that inhibition of reproduction by adult worms was impaired in some repeatedly treated communities in contrast to an ivermectin-naïve community. Subsequent repopulation of skin by microfilariae was faster than expected even after considering the inter-study variability of the (also ivermectin-naïve) meta-analysis. A model for repopulation rates was fitted to microfilarial temporal profiles after treatment for each person examined in one of the communities (treated for 10 years) to quantify the level of inter-individual variability in parasitological response. Ivermectin resistance is common in veterinary parasites and has a genetic basis associated with selection on ATP-binding cassette (ABC) transporters (e.g. P-glycoproteins) and β-tubulin. *Onchocerca volvulus *samples from communities in Ghana and Cameroon that have received many treatments have been found to have significant changes in similar genes (β-tubulin, P-glycoproteins and other ABC transporters) compared with worms isolated from treatment-naïve subjects or the same subjects prior to treatment [16,17]. These genetic changes should be useful markers for ivermectin resistance monitoring. Genetic and parasitological data can be integrated into mathematical models to assess the epidemiological consequences of anthelmintic resistance, identify optimal host characteristics and parasitic stages to sample, and assist in the development of intervention strategies to delay or manage resistance.

### Alternative Chemotherapy, drug discovery and development

#### Antibiotic therapy

New and alternative treatments for filariasis are therefore urgently needed. The break-through of using antibiotics to target the bacterial symbiont of the parasite has identified a novel treatment and target that offers a superior therapeutic alternative to current anthelminthic drugs [18-21]. The rationale for this novel treatment is the antibiotic targeting of *Wolbachia *– a bacterial endosymbiont of filarial parasites essential for worm development, fertility and survival and inducer of inflammatory disease pathogenesis. A series of field trials against onchocerciasis and lymphatic filariasis have demonstrated that 4–8 week courses of the antibiotic doxycycline deplete the bacteria and result in the long-term sterility and most importantly death of adult worms [18-21].

A series of field trials on onchocerciasis in Ghana began with a regimen of daily treatment with 100 mg doxycycline for 6 weeks, followed by ivermectin 4 months later, which resulted in depletion of *Wolbachia *and female worm sterility for 18 months in all worms except for a few that were acquired after doxycycline treatment [21-23]. The data allowed the conclusion that this effect was due to doxycycline alone [22]. In a more recent, placebo controlled trial, treatment with a daily dose of 200 mg, either for 4 or 6 weeks (again followed by ivermectin), also lead to sterility of female worms and more importantly to a macrofilaricidal effect of 50 or 60%, respectively, observed at 21–27 months after treatment [24]. If one subtracted those worms that had been newly acquired in the interval after doxycycline treatment, the macrofilaricidal rate would have been approximately 10% higher. The proportion of dead female worms in ivermectin-only treated patients who had received placebo instead of doxycycline remained at levels equivalent to those found in onchocercomas from untreated patients (around 15%). The macrofilaricidal effect of doxycycline appears to take >1 year to manifest accounting for the lack of this activity in earlier reports. In support of this, another study using 100 mg/day of doxycycline for 5 weeks (without ivermectin) also led to sterility and a 50% macrofilaricidal effect after 21–27 months [25].

The 'slow-kill' outcome of doxycycline therapy has several advantages including the elimination of the inflammatory inducing bacteria [26,27] and avoidance of potential adverse reactions to nematode products associated with a rapid-kill as observed in loiasis co-infection [3].

The current major obstacles to the use of antibiotic therapy in control of filariasis is the length of treatment, which is considered to be logistically incompatible with the community-directed treatment strategies used for filariasis control and contraindication for children >9 and pregnancy. Shorter regimes using combinations of existing antibiotics effective against *Wolbachia *require testing to establish the minimum effective regime for integration into existing control programmes to meet the programmatic aims of eliminating filariasis or be used in more restricted situations, such as during the 'mop-up' phase and the end of programmes, in the event of the emergence of resistance or poor responsiveness to existing drugs or in individuals co-infected with *Loa loa *and at risk of severe adverse reactions to ivermectin. Definition of the minimal effective regime will also provide an important advance in the treatment options for individual cases outside of controlled areas.

In the case of communities co-infected with *L. loa*; the risk of severe adverse events following ivermectin therapy in individuals with high intensity of *L. loa *microfilaraemia precludes the use of ivermectin in many of the forested regions of APOC. The use of doxycycline in these communities has been determined in two trials in Cameroon. 1) A placebo-controlled trial of doxycycline with or without ivermectin in areas of low loaisis co-endemicity, and 2) a trial of community-directed delivery of doxycycline alone in communities with high prevalence of loaisis. The outcome of these trials, which is currently under analysis, should reveal the suitability of these approaches as an alternative therapy for onchocerciasis in areas of loiasis co-endemicity.

### Anti-*Wolbachia *(A-WOL) Drug Discovery and Development

In an attempt to translate Anti-*Wolbachia *therapy into a public health tool the A-WOL consortium has been established through funding from the Bill and Melinda Gates Foundation to find new drugs active against *Wolbachia *that improve upon existing antibiotic therapy.

The principal goal is *the establishment of anti-symbiotic chemotherapy directed against Wolbachia compatible with mass drug administration (MDA) programmes for human filariasis*. The expected health outcome would be an alternative treatment for human filariasis to ensure that the long-term goals of global elimination of the public health problems are achieved. A secondary goal is *the establishment of anti-symbiotic chemotherapy suitable for a more restricted use, planned for an interim phase in which there is yet no drug or drug combination available that is compatible with MDA usage*. For example, in the event of the emergence of drug-resistance to existing treatment in limited populations or as an alternative for communities co-endemic for loiasis.

In order to achieve these goals A-WOL has identified four major objectives to address the discovery and development of new drugs and regimes against *Wolbachia *(see Appendix 1). The strategy is intended to cover; 1) the optimization of regimes of drugs with known activity against *Wolbachia *(doxycycline and rifampicin, [28,29]) for integration into existing MDA programmes within 5 years, 2) the evaluation of anti-*Wolbachia *drugs already licensed for human use, which could enter control programme deployment without the need for extended toxicity and regulatory processes and 3) the identification of novel drugs and compounds identified through high-throughput screening and target discovery to provide a portfolio of lead candidates in a product pipeline, which could be used in future control and treatment approaches.

Progress during the first 12 months of the programme includes the discovery of novel tetracyclines with improved *in vitro *and *in vivo *efficacy to doxycycline, novel classes of antibiotic and other drugs active against *Wolbachia *and validated targets for novel drug design, which provide encouragement that the goals of the programme will be achieved.

### WHO-TDR: Anthelminthic Drug Discovery and Development

Drug discovery and development activities for onchocerciasis continue under the TDR helminth drug initiative. Drugs from a variety of sources are screened against *Onchocerca lienalis *microfilariae and *O. gutturosa *adult male worms *in vitro *and *O. lienalis *microfilariae in mice. Hits from these screens are then tested against *Brugia pahangi *in jirds and dogs. From 2006/7 some 5000 compounds have been screened *in vitro *producing ~100 compounds for follow-up and around 6 lead compounds for optimization. In addition, cyclooctadepsipeptides such as emodepside, show species-dependent activity against filarial nematodes. Variability in sensitivity to larval and adult stages is reported for *Acanthocheilonema viteae*, *L. sigmodontis *and *B. malayi *[30], but it remains an effective microfilaricide in all three species [31].

Another drug being developed for control programme use is moxidectin (Wyeth). A partnership between endemic countries, APOC, TDR and Wyeth is undertaking the development required to determine whether moxidectin has the efficacy and safety not only to eliminate onchocerciasis as a public health problem but to eradicate onchocerciasis. The development has progressed through studies in healthy volunteers to an ongoing Phase II hospital-based trial of up to 192 *O. volvulus *infected subjects which will complete the 18 months efficacy follow up at the end of 2009. Based on the safety and microfilaricidal efficacy data obtained in this trial to date, initiation of a large phase III trial is planned. This will be followed by a study to determine a safe dose in children before application for regulatory agency approval. Data from community studies on safety, efficacy and effect on transmission will be obtained before an expert meeting to assess whether moxidectin can be recommended for use in control programmes by 2014.

### Vector Control

Vector control was a major component of the onchocerciasis control programme (OCP) and was based on routine aerial application of larvicides over a period of more than 14 years. This is an expensive method of treating the river, but was considered essential due to the vast area within the project. Since OCP was completed, vector control has not been considered feasible or cost-effective in the APOC countries, except for a few small isolated foci in 'special intervention zones' (SIZ).

In Ghana, vector control has virtually ceased, except for limited activities associated with the Bui Dam construction, but in Cameroon, temephos has been applied across the full width of the Sanaga river, at selected sites using local boats. Three applications were made ideally at 7 day intervals, with adult blackflies monitored on Perspex sheets covered with a sticky substance, to determine when a subsequent series of larvicide treatments should be applied to keep adult fly populations below a threshold. Other insecticides used in a rotation will be needed to minimise the risk of resistance. Dosage applied was calculated according to WHO recommendations.

These control operations were required where areas of tropical rain forest along the Nyong and Sanaga rivers were de-populated due to the high incidence of onchocerciasis and the constant irritation caused by the bites of the vector *Simulium *spp. The latter affects people's skin and prevents working a full day, so agricultural production has declined. The severity of black flies is considered to have increased and this may be partly due to hydroelectric schemes along the Sanaga River.

So far larval control has been confined to major rapids on the Sanaga, but to treat narrower rivers, larvicide could be applied using motorised hydraulic knapsack sprayers fitted with boomless nozzles to project the insecticide across the river.

To minimise treatment of rivers, research is need to determine whether adult blackflies can be lured by an attractant to insecticide treated screens as used in tsetse control, and whether insecticide treated over-garments or bandana at the wrists and ankles could reduce the impact of the flies on field workers. Further research is needed to improve monitoring and establish thresholds that determine when control is needed.

In Ghana on-going transmission shows that operational research remains critical to sustain gains of the OCP and improve onchocerciasis control. A closely monitored area-wide blackfly management programme could significantly reduce the populations of blackflies, and thus allow agricultural productivity to increase in West Africa. By integrating vector management with drug treatment, such a programme could lead to better use of the drugs by those affected by onchocerciasis. However, capacity building is needed to increase local knowledge of vector ecology, sampling procedures and control techniques.

## Conclusion

The outcome of the meeting was a list of priorities for research (Appendix 2). Some of these areas are ongoing and funded, but a number of important areas require additional support and renewed effort. Failure to grasp the importance and urgency of addressing these activities runs the serious risk of undoing much of the benefits and achievements of past and current control programmes. A unanimous outcome was the belief that, where possible, this research should be undertaken by African scientists within endemic countries. Sufficient capacity building and funding for such activities are urgently required if the aims of the Yaoundé declaration [32] are to be realised and the future control of onchocerciasis is to be secured.

## Competing interests

The authors declare that they have no competing interests.

## Authors' contributions

MT compiled the report from contributions to each section written by each of the other authors. All authors approved the final draft of the report.

## Appendix 1

A-WOL has four main objectives:

1) Regime Refinement – To address this we will carry out two phase II field trials in Ghana on *O. volvulus *and *W. bancrofti *to test combinations of doxycycline with rifampicin at low doses to achieve shorter regimens to define the optimal regime using drugs already licensed for human use.

2) Assay development – To develop *Wolbachia*-cell line assays compatible with combinational high throughput screening (cHTS) technologies and as a novel tool to screen existing and novel compounds for anti-symbiotic activity.

3) Library Screening – To screen novel derivatives of tetracycline (TC), large diversity libraries (~100 K compounds) and combinations of existing drugs against *Wolbachia*.

4) Target Discovery – To identify targets and inhibitors of key enzymatic and metabolic pathways predicted from *Wolbachia *genomic annotation. To use bioinformatics to identify the 'essential gene set' of *Wolbachia *and multi-step computational approaches to identify potential drug targets and develop a surrogate screening system in *E. coli*. To use aptamer technology to identify inhibitors of novel drug targets to incorporate into HTS.

## Appendix 2

### Research priorities

#### Mapping responsiveness to ivermectin

Information on the extent and geographical range of ivermectin responsiveness is needed through:

- Spatial and longitudinal monitoring of epidemiological, parasitological, vector and clinical responses

- Investigation of parasite genetics in relation to drug susceptibility and the development of genetic markers to quantify connectivity between parasite populations

#### Alternative/complementary/combined control strategies

- Improved integration of existing strategies of chemotherapy and vector control

- Re-evaluation of control strategies for drugs with macrofilaricidal or permanent sterilizing activity

- Continue development of macrofilaricidal drugs and approaches to deplete the parasites of *Wolbachia* endosymbionts

#### Diagnostic tools

- To develop specific, rapid and less invasive tools to improve mapping and provide prospective tools to define control programme endpoint

#### Vector control

- Evaluate new methods of blackfly trapping for monitoring and to reduce fly density

- Optimizing techniques to apply larviciding

- Develop research on blackfly attractants, reducing human-vector contact, improved knowledge of vector-parasite combinations

- Consider role of Community-directed activities for vector control

#### Quantitative population biology / Mathematical modelling

To assist in the planning of activities for:

- Identification and optimisation of indicator groups (in the area of diagnostics and programme monitoring)

- Monitoring of resistance (detection and quantification of genetic markers)

- Exploring different control scenarios, combinations of control measures, timings of applications, etc. that can also delay spread of drug resistance

- Vector biology, quantification of vector competence, vector population dynamics, insecticide resistance

Capacity building in all the areas above
